# Defying the Odds: A Case Report of ACTG2-Related Megacystis-Microcolon-Intestinal Hypoperistalsis Syndrome With Complete Recovery

**DOI:** 10.7759/cureus.84449

**Published:** 2025-05-20

**Authors:** Naila Almoosa, Nagham Alshehabi, Farheen Khan, Mira Elmiaari, Alia Magzoub, Maysa Saleh

**Affiliations:** 1 Pediatrics, Al Jalila Children’s Specialty Hospital, Dubai, ARE; 2 General Pediatrics, Al Jalila Children’s Specialty Hospital, Dubai, ARE

**Keywords:** actg2, megacystis-microcolon-intestinal hypoperistalsis syndrome, parenteral nutrition, pseudo-obstruction, visceral myopathy

## Abstract

Visceral myopathy is a rare and complex congenital disorder primarily impacting the gastrointestinal and urologic systems. Among its manifestations, megacystis-microcolon-intestinal hypoperistalsis syndrome (MMIHS) represents the most severe form. Typically, this condition has a poor prognosis, with all reported cases necessitating lifelong parenteral nutrition, frequent surgical interventions, and intermittent catheterization. We present the case of a three-month-old female infant with a history of *ACTG2*-related MMIHS who presented with oliguria, emesis (non-bilious to bilious), and fever of 38.9°C over a two-day period. A gastrointestinal panel identified enteroaggregative *Escherichia coli*, and imaging revealed distended bowel loops without evidence of ischemia, microcolon, or obstructive lesions. The distended bladder required temporary catheterization, which was later removed. Initial parenteral nutrition was discontinued after eight days as the infant tolerated oral feeds well. The hospitalization was further complicated by a urinary tract infection and thrombocytosis, but the patient was ultimately discharged on full oral feeds with spontaneous urine output. This case contrasts the typical poor prognosis of visceral myopathy and MMIHS with a favorable outcome. The patient, who led an asymptomatic life until three months of age, avoided surgical interventions, long-term parenteral nutrition, and intermittent catheterization, interventions commonly required for MMIHS. After identifying an underlying infection that triggered pseudo-obstructive symptoms, symptomatic management was implemented. Once the infection resolved, no further interventions were necessary. This approach was not only cost-effective but also reduced the physical and emotional burden of the family, underscoring the importance of early diagnosis and targeted treatment for positive outcomes.

## Introduction

Visceral myopathy is a rare and poorly understood disorder that primarily affects the gastrointestinal and urologic systems, leading to dysfunction of smooth muscle and the enteric nervous system. This condition is often associated with a wide range of symptoms, including feeding difficulties, chronic constipation, bowel obstruction, and urinary retention. The spectrum of severity can vary, with megacystis-microcolon-intestinal hypoperistalsis syndrome (MMIHS) representing the most severe phenotype. MMIHS, also known as Berdon syndrome, is a congenital disorder often caused by mutations in the *ACTG2* gene, which encodes gamma-actin, a key protein involved in smooth muscle function. This syndrome is characterized by impaired motility in the gastrointestinal tract and bladder, leading to progressive dysfunction and requiring long-term interventions [1].

The diagnosis of visceral myopathy, and particularly MMIHS, is complex due to its nonspecific clinical presentation, which often overlaps with more common gastrointestinal and urologic disorders. Symptoms such as feeding intolerance, delayed gastric emptying, and urinary retention can be subtle, especially in neonates and infants, and may not be immediately recognized as part of a rare genetic syndrome [1]. Genetic mutations in *ACTG2*, particularly recurrent arginine substitutions, have been identified as the primary cause of visceral myopathy, influencing disease severity and burden [2]. However, the pathophysiology remains complex, as it involves not only smooth muscle dysfunction but also potential abnormalities in the enteric nervous system, further complicating both diagnosis and treatment [2].

The prognosis for MMIHS is typically poor, with most affected individuals requiring lifelong parenteral nutrition, multiple surgical interventions, and intermittent catheterization. However, early diagnosis and appropriate management strategies, including nutritional support, symptom control, and surgical interventions, can improve outcomes significantly [3]. Recent advances in genetic testing and a deeper understanding of *ACTG2*-related disorders have opened the possibility for more timely diagnoses and better individualized care plans, which may improve quality of life and survival rates for affected individuals [1]. This case report aims to highlight the potential for favorable outcomes with early detection and intervention, offering a challenging perspective on the traditionally poor prognosis associated with MMIHS.

## Case presentation

A three-month-old female presented to the emergency department of a tertiary pediatric hospital. She was born at 37 weeks of gestation via cesarean section in India, with a birth weight of 2.3 kg. The cesarean delivery was prompted by fetal bladder distention (megacystis) and borderline polyhydramnios detected on antenatal ultrasound.

The patient presented with a two-day history of vomiting, six times per day, which was initially nonbilious and nonbloody. On the day of her visit, she vomited five times, the fifth episode being bilious but non-projectile. The vomiting was postprandial and consisted mostly of milk content. Additionally, she experienced a fever for one day, with a maximum recorded temperature of 38.9°C, which responded to antipyretics, and a decrease in urine output over the past two days. Stool changes included light yellow, mucus-filled stools, and one episode of green stool. Despite these symptoms, the mother reported that the patient was still feeding well.

The patient had a history of prenatal concerns, including megacystis and borderline polyhydramnios, which led to a cesarean delivery. After birth, she spent 10 days in the neonatal intensive care unit due to the need for frequent catheterization. Whole-exome sequencing revealed a heterozygous autosomal dominant mutation in the *ACTG2* gene, leading to the diagnosis of MMIHS. Before this presentation, the patient had been growing well, breastfeeding without difficulty, and passing urine spontaneously. She had no prior hospitalizations or health concerns, and her vaccination records were up-to-date.

On physical examination, the patient appeared pale, dehydrated, and fatigued, with a distended, tense, and tender abdomen. Her vitals showed tachycardia at 169 beats per minute, a fever of 38.9°C, and tachypnea at 42 breaths per minute. Capillary refill time was delayed at 2-3 seconds.

**Figure 1:**
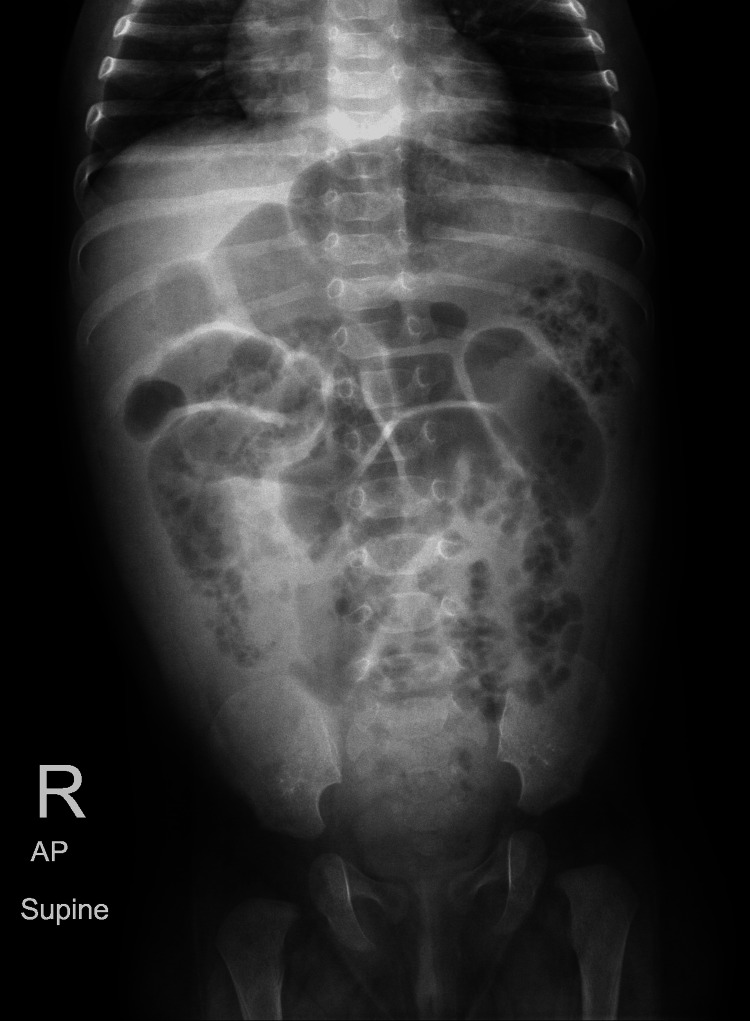
Abdominal X-ray. Moderate gaseous distension of the stomach and the bowel loops are noted in the center of the abdomen with a “soap-bubble” appearance of the nondistended bowel loops in the periphery with rectal air.

**Figure 2:**
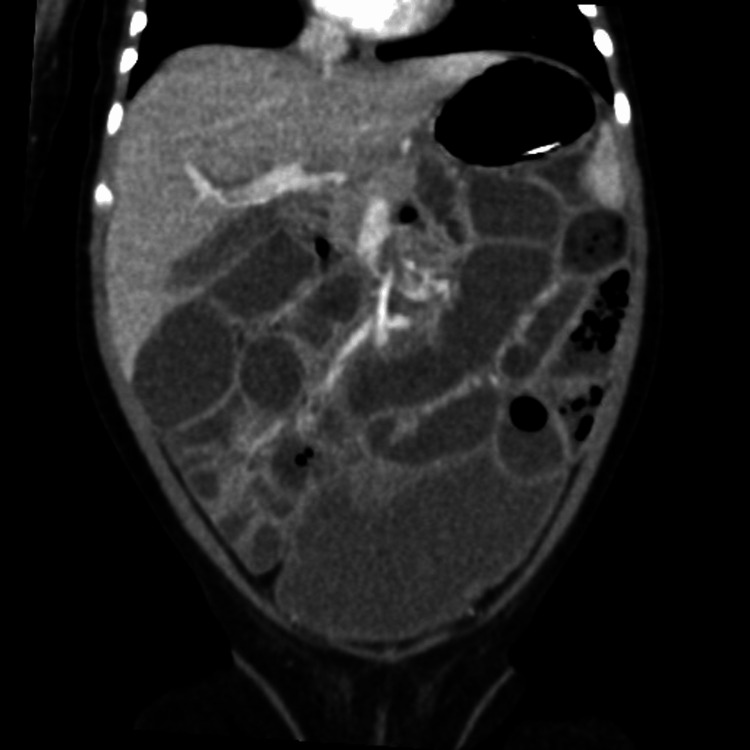
CT of the abdomen (coronal view).

**Figure 3:**
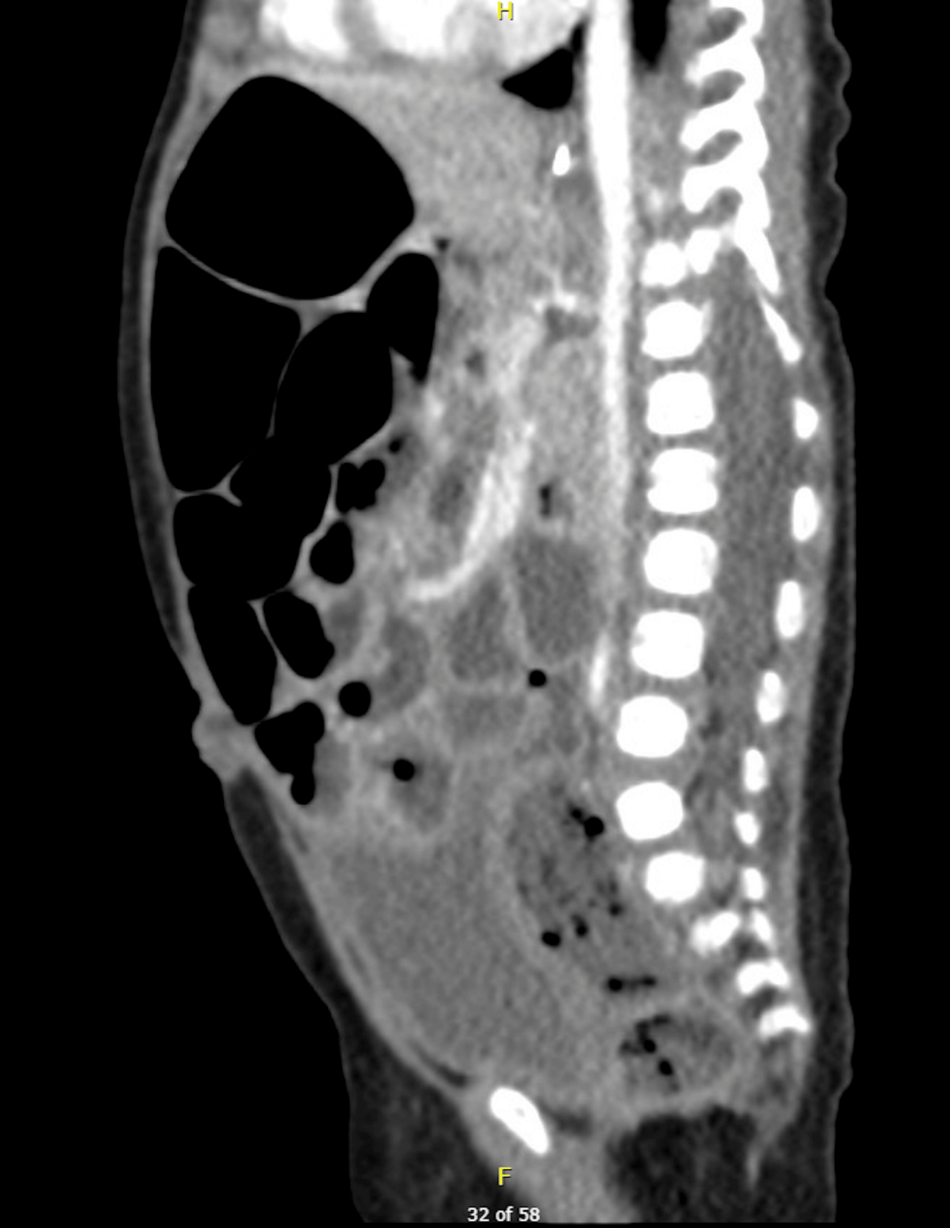
CT of the abdomen (lateral view). Sagittal views of the patient’s abdominal CT showing a distended bladder and stomach, with a feeding tube in situ. No evidence of pneumatosis intestinalis or pneumoperitoneum. No obvious transition zone or signs of malrotation.

**Figure 4:**
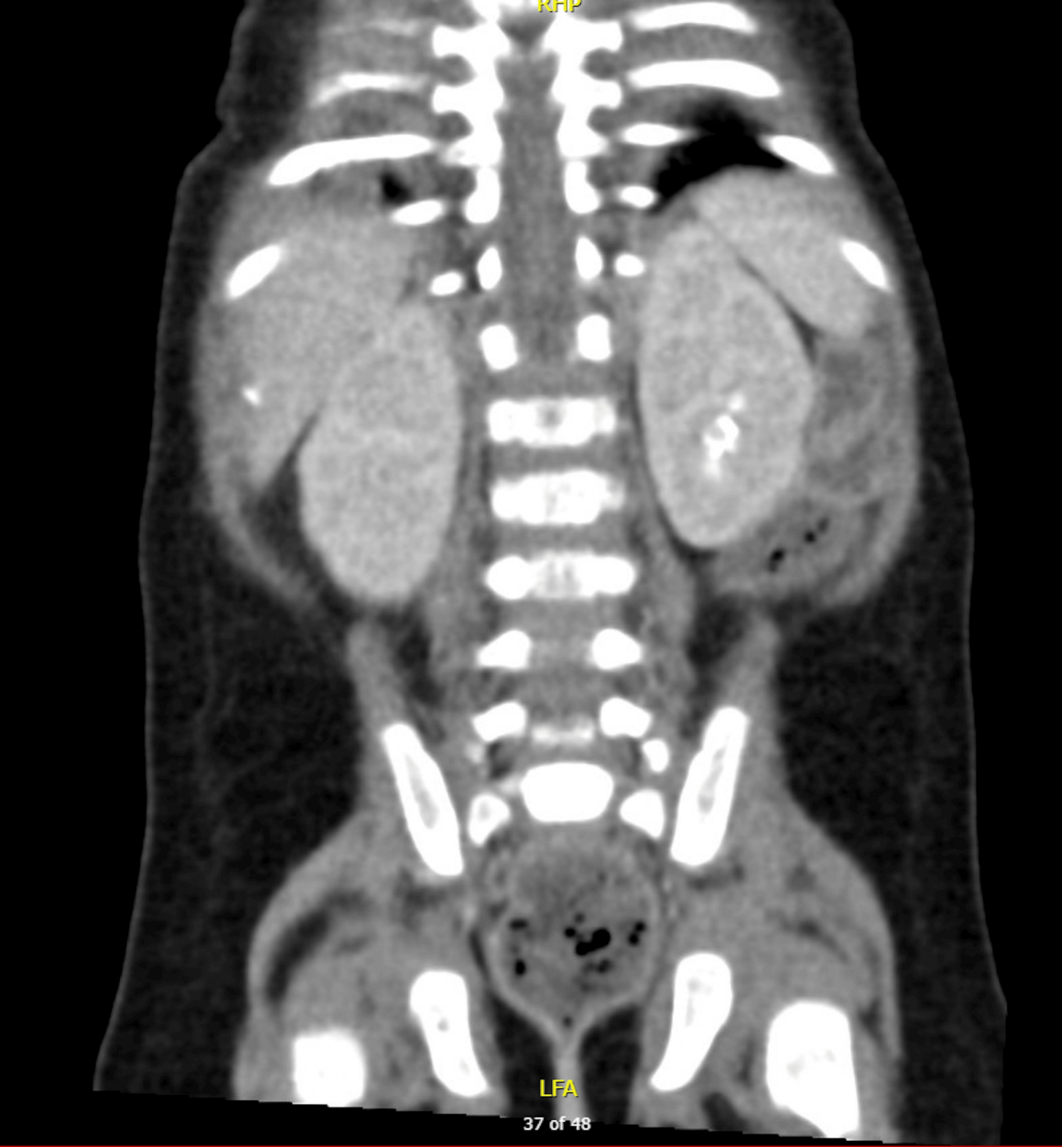
CT of the abdomen (bladder view). Coronal views of the patient’s abdominal CT showing a distended bladder and stomach.

Given the presenting symptoms of vomiting and abdominal distention, initial differentials included small bowel obstruction and pyloric stenosis. To rule out these conditions, an initial abdominal X-ray and ultrasound were conducted. The X-ray (Figure 1) revealed a soap-bubble appearance of the bowel loops but no signs of intestinal obstruction, while the abdominal ultrasound showed no evidence of pyloric stenosis. As the initial imaging did not support these differentials and was not fully consistent with the clinical presentation, a surgical consult was obtained. While no immediate surgical intervention was required, a repeat abdominal X-ray raised concern for bowel ischemia. In response, intravenous metronidazole was administered. A subsequent CT scan (Figures 2-4) revealed distended small and large bowel loops, but no obstructive lesions, microcolon, pneumatosis intestinalis, or pneumoperitoneum. The bladder was also noted to be distended, prompting the insertion of an indwelling catheter on the second day of admission to facilitate drainage and monitor urine output. Initial laboratory findings are presented in Table 1.

**Table 1:** Initial laboratory findings.

Test	Result	Reference range	Interpretation
White blood cell (WBC) count	5.1 × 10³/μL	6.0–18.0 × 10³/μL	Leukopenia
Platelet count	1,022 × 10³/μL	200–550 × 10³/μL	Thrombocytosis
Red blood cell count (RBC)	3.6 × 10⁶/μL	4.10–5.30 × 10⁶/μL	Low RBC count
C-reactive protein (CRP)	64 mg/L	0–5 mg/L	Elevated CRP
Gastrointestinal panel	Positive for *Escherichia coli*	Not detected (negative)	Positive for enteroaggregative *Escherichia coli*

The patient was first resuscitated with a 0.9% saline bolus (20 mL/kg) and kept nil per os, with a nasogastric tube inserted for decompression. The patient was initially admitted as a case of suspected diagnosis of sepsis and *Escherichia coli* gastroenteritis. Empiric intravenous antibiotics were started due to suspected sepsis. The family disclosed the patient’s underlying MMIHS on day two, which prompted a shift in management toward addressing the exacerbation of MMIHS, worsened by the *E. coli* infection, leading to pseudo-obstruction. Gastroenterology recommended supportive care and nutrition.

On day three, urine cultures confirmed extended-spectrum beta-lactamase-producing *Klebsiella*, and empirical antibiotics were switched to piperacillin-tazobactam (Tazocin). The patient developed hypokalemia, which was corrected, and total parenteral nutrition (TPN) was initiated for eight days. By day five, oral feeding was tolerated, and the indwelling catheter was removed as urine output normalized. The patient completed a 10-day course of piperacillin-tazobactam.

A repeat urine culture was positive for *Candida albicans*, and fluconazole was started. After a third repeat culture was negative, fluconazole was discontinued after two days.

**Figure 5:**
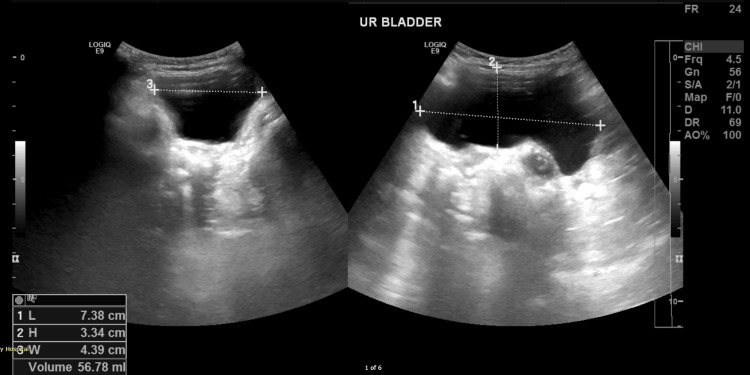
Ultrasound of the bladder. The urinary bladder before discharge is well distended (56 mL), showing normal wall thickness and no obvious intraluminal abnormality.

Given the complexity of MMIHS, a multidisciplinary team was involved in the patient’s management. The gastroenterology team managed the pseudo-obstruction and provided nutritional support. Cardiology conducted an echocardiogram, which showed normal heart function despite the potential risks associated with *ACTG2*-related conditions. Ophthalmology observed mydriasis, but no ocular abnormalities were found. Lastly, a repeat ultrasound of the kidney-ureter-bladder was performed and showed normal results before discharge (Figure 5).

The patient was discharged in stable condition, tolerating oral feeds, and prescribed trimethoprim/sulfamethoxazole prophylaxis. Ongoing supportive care for future pseudo-obstruction episodes was recommended, with a comprehensive follow-up plan involving all relevant specialties. Unfortunately, the patient was a no-show for her follow-up appointments; therefore, we are unsure regarding her progress at this time.

## Discussion

Visceral myopathy, primarily due to *ACTG2* gene mutations (*ACTG2*-related myopathy), presents with varying phenotypes, including MMIHS, chronic intestinal pseudo-obstruction, and multisystemic smooth muscle dysfunction syndrome (MSMDS) [1,2]. These conditions share significant genotypic and phenotypic overlap.

MMIHS is a rare congenital disorder characterized by an enlarged bladder, reduced intestinal motility, and a small colon, with approximately 450 reported cases [3,4]. It mainly affects females (female-to-male ratio: 2.3-4:1) [4,5], with 44% of cases caused by *ACTG2* mutations (autosomal dominant). Autosomal recessive and familial cases (12%) related to parental consanguinity cases involve mutations in *LMOD1*, *MYH11*, *MYL9*, and *MYLK* ‎[4-7].

*ACTG2* mutations disrupt smooth muscle contraction signaling, leading to histological changes such as immature ganglion cells and thin muscle layers [8,9]. Overall, 25% of diagnoses often occur prenatally on prenatal ultrasound due to fetal megacystis, polyhydramnios, or hydronephrosis [10-12]. In infancy, diagnosis is triggered by gastrointestinal pseudo-obstruction and urinary retention, presenting commonly with abdominal distention, bilious vomiting, and failure to pass meconium within 24 hours of life [10]. Milder cases may present later with intussusception or volvulus [13,14].

Imaging typically shows gastrointestinal dilation, microcolon, megabladder, and hydronephrosis. Multisystemic manifestations may include deafness, blindness, hydrocephalus, and cryptorchidism [15-18]. Specific signs include dilated pupils (*MYL9*-related) and vascular smooth muscle dysfunction (*MYH11*-related) [10-19].

Of note, our case had an exaggerated mydriatic response, which is usually associated with *MYL9* mutations, and lacked a microcolon despite having both decreased peristalsis and megacystis on imaging, as well as a clear genetic diagnosis. Traditionally, the presence of a microcolon distinguished prune belly syndrome from MMIHS, but this is no longer reliable. Several MMIHS cases without a microcolon have been reported, including seven with *ACTG2* mutations [15,20,21]. Some surviving cases required daily catheterization or TPN [15,21], and one case lacked megacystis altogether [22]. This suggests variable expressivity in MMIHS, possibly warranting classification under the broader *ACTG2*-related visceral myopathy spectrum.

Management of MMIHS requires a multidisciplinary approach, involving gastroenterology, urology, cardiology, genetics, ophthalmology, and pediatrics [3,10]. Outcomes improve when care is provided at specialized centers, particularly for patients requiring parenteral nutrition [6]. Around 50% of MMIHS cases undergo surgical interventions, mainly for gastrointestinal decompression (e.g., gastrostomy or ileostomy), although the benefit of these procedures is debated [6].

A recent review of urological management highlighted the importance of bladder protocols, such as clean intermittent catheterization or vesicostomy, and early specialist involvement in preventing urinary tract infections and kidney dysfunction [23]. Vesicostomy is performed in 25-30% of cases [5], and long-term intermittent catheterization is often necessary due to bladder dysfunction. However, in contrast, our case demonstrates a more favorable outcome, with the patient able to pass urine spontaneously and avoid urinary catheterization after resolving a triggering infection.

Historically, MMIHS has had a poor prognosis, often resulting in death from sepsis, malnutrition, or complications related to TPN [5,7]. However, survival rates have improved, with 10- and 20-year survival rates reaching 100% and 86%, respectively, due to advances in treatment, including transplantation [6]. These improvements are linked to timely referrals, better TPN management, and infection prevention [6].

Our case contrasts the typical poor prognosis of MMIHS by showcasing a favorable outcome with conservative management. The patient, who had been asymptomatic until three months of age, avoided surgery, long-term TPN, and intermittent catheterization. After identifying and treating an underlying infection that triggered pseudo-obstruction, no further interventions were necessary. This case highlights the value of early diagnosis, understanding the disease’s underlying pathology, and targeted treatment, leading to a cost-effective and less burdensome approach with better outcomes for both the patient and family.

## Conclusions

This case highlights a unique presentation of MMIHS. Despite the generally poor prognosis associated with this disorder, our patient demonstrated an unexpectedly favorable outcome. Notably, the patient did not require long-term parenteral nutrition, surgical intervention, or intermittent catheterization, which are typically necessary in most cases. Furthermore, this case underscores the importance of early diagnosis, multidisciplinary management, and supportive care in potentially mitigating the severity of the disease and improving quality of life. As more is being discovered about the varied presentations of MMIHS, this case serves as a hopeful reminder that recovery is possible even in severe congenital conditions such as visceral myopathy.
